# *Thraumata*, a new genus from South America with description of a new species from Peru (Lepidoptera, Noctuidae)

**DOI:** 10.3897/zookeys.867.28728

**Published:** 2019-07-31

**Authors:** Paul Z. Goldstein, Alberto Zilli

**Affiliations:** 1 Systematic Entomology Laboratory, USDA, National Museum of Natural History, E-502, P.O. Box 37012, MRC 168, Washington, DC 20013-7012, USA United States Department of Agriculture Washington United States of America; 2 Life Sciences, Insects Division, The Natural History Museum, Cromwell Road, London SW7 5BD, UK The Natural History Museum London United Kingdom

**Keywords:** *
Phuphena
*, Noctuidae, owlet moths, Peru, systematics

## Abstract

*Thraumata***gen. nov.** is described to accommodate three South American species, two previously placed in *Phuphena* Walker, 1858, namely *Thraumata petrovna* (Schaus, 1904), **comb. nov.** and *Thraumata subvenata* (Schaus, 1914), **comb. nov.**; and one, *Thraumata peruviensia***sp. nov.**, newly described from Peru. Although the larval biology is unknown, these species share several features that suggest their placement in Eriopinae and, as a consequence, a potential association with ferns (Pteridophyta) as larval host plants.

## Introduction

*Phuphena* Walker, 1858 appears to represent a polyphyletic assemblage that includes a morphologically homogeneous, putatively monophyletic core group of 10 described species: *P. cilix* (Druce, 1898), *P. constricta* Dognin, 1912, *P. diagona* Hampson, 1908, *P. multilinea* Schaus, 1911, *P. obliqua* (Smith, 1900), *P. parallela* (Hampson, 1904), *P. proselyta* Schaus, 1921, *P. transversa* (Schaus, 1894), *P. tura* (Druce, 1889), *P. zelotypa* Schaus, 1911 and the type species *P. fusipennis* Walker, 1858. Outside this assemblage are *Phuphena costata* Schaus, 1914, which will be treated elsewhere, and two of the species treated here, *P. petrovna* (Schaus, 1904) and *P. subvenata* Schaus, 1914. Of the 15 available names for species in the genus *Phuphena*, William Schaus described eight in six publications between 1894 and 1921. Three of these species he originally placed in other genera, in some cases denoting the generic name with a question mark, and among these was *P. petrovna* (Schaus, 1904), which Schaus (1904) described in *Leptina* Guenée 1852 nec Meigen 1830 (= *Baileya* Grote, 1895) (Nolidae). In the course of revising *Phuphena* we identified *P. petrovna*, *P. subvenata*, and an undescribed species similar to *P. petrovna* as missing features diagnostic of typical *Phuphena* species while sharing a number of conspicuous features not observed elsewhere in the genus. Our primary purpose here is to describe the new species and a new genus *Thraumata* gen. nov. to accommodate all three species, and as a possible addition to the Eriopinae, now under study. *Phuphena* is currently recognized within the Phosphilini and, although this placement will be treated more thoroughly in a separate revision of *Phuphena*, we address a number of features here that seem to contradict it. We further discuss several characters that bear both on the placement of both *Phuphena* and *Thraumata*, and include a diagnosis of each genus.

## Materials and methods

Genitalic preparations follow Clarke (1941) in part and Lafontaine (2004), but staining with chlorazol black and slide-mounting in Euparal. Vesicae were everted with a microsyringe prior to fixation. Dissections followed either an overnight room-temperature soak or a brief 15-minute heated soak in supersaturated sodium hydroxide solution and were examined under dissecting microscopes prior to slide-mounting. Wing preparations followed the procedure modified from Jaeger (2017): following an overnight soak in a small stender dish with enough 50% EtOH to cover the wings and 10 drops of 6% NaCl, ethanol and bleach were added as needed, depending on the ease with which scales could be cleared. Wings were then stained overnight in Eosin Y. Images were taken using Microptics and Visionary Digital imaging systems and images were manipulated with Adobe Photoshop and Illustrator (Adobe Systems, Mountain View, CA). Images of *T. petrovna* vesicae were taken in glycerin, held in a sectioned plexiglass cylinder affixed to a slide. Measurements were made with the aid of an ocular micrometer. Forewing (FW) length was measured from the center of the axillary area up to the apex of the forewing. Terminology generally follows Forbes (1954) and Lafontaine (1998, 2004).

Prior to dissection, the abdomens of several specimens underwent a 24-hour soak in a 10% proteinase K solution in the Laboratory of Analytical Biology at USNM. DNA extractions and partial DNA barcodes were then generated at the Biodiversity Institute of Ontario.

Exemplars used for comparative purposes included specimens of generotypes within Phosphilini and Eriopinae, including *Phuphena*, *Phosphila* Hübner, 1818, *Acherdoa* Walker, 1865, and *Callopistria* Hübner, [1821].

### Repository abbreviations

The following abbreviations refer to collections from which specimens forming the basis of this study originated:

**AMNH** American Museum of Natural History, New York, USA

**NHMUK** [formerly, BMNH] The Natural History Museum, London, UK

**USNM**National Museum of Natural History [formerly, United States National Museum], Washington, District of Columbia, USA

## Systematics

### Phuphena

Taxon classificationAnimaliaLepidopteraNoctuidae

Walker, 1858

8dec755a-e41c-5e8e-99a6-5011d0af8368

#### Type species.

*Phuphena fusipennis* Walker, 1858 by monotypy.

#### Diagnosis.

Species of *Phuphena* diverge with respect to wing pattern, but most share a bounded medial area. Orbicular and reniform stigmata sometimes absent or indistinct; outer forewing margin smooth in the majority of species but crenulate in *cilix*. Hindwing upperside coloration uniform. Male genitalia of *Phuphena* distinct, the valvae uniquely reduced, with no evidence of clasping architecture, narrow for most of their length, and in most species swollen apically to form a knob-like cucullus, giving them a club-shaped appearance, with a corona comprising an unorganized arrangement of setae. Vinculum V-shaped, slightly truncate in some species, and extending below the base of the valves for a distance equal to or greater than the width at its broadest point. The phallus is asymmetrically sclerotized, often with a patch of scale-like cornuti, and the vesica without well-developed cornuti or diverticula, but with a corresponding ventro-distal strip of scale-like cornuti. Basal abdominal brushes present but not visibly subdivided as in *Callopistria* Hübner, [1821] (type species: *Noctua juventina* Stoll, 1782); a smaller pair on A8, and eversible saccular hair pencils completely absent. Female genitalia simple, the corpus bursae lemon-shaped, with no appendix or signa, the ductus seminalis attached apically, to the distal (anterior) end of the corpus. This distinguishes *Phuphena* from all *Thraumata* spp. and from *Callopistria juventina*, in which the ductus arises from the proximal (posterior) end of the corpus, but this character varies among other *Callopistria* species.

### Thraumata gen. nov.

Taxon classificationAnimaliaLepidopteraNoctuidae

b2c8c88b-90d3-517a-8677-d0c042d22814

http://zoobank.org/EFC5C009-2693-43AB-BDF5-3507B02E5FB3

#### Type species.

Leptina ? petrovna Schaus, 1904, by present designation.

#### Etymology.

*Thraumata* is the nominative neuter plural form of the Greek *θραυμα*, meaning shard, fragment, or potsherd, in reference to the fragmented appearance of the forewing pattern.

**Figures 1–9.**
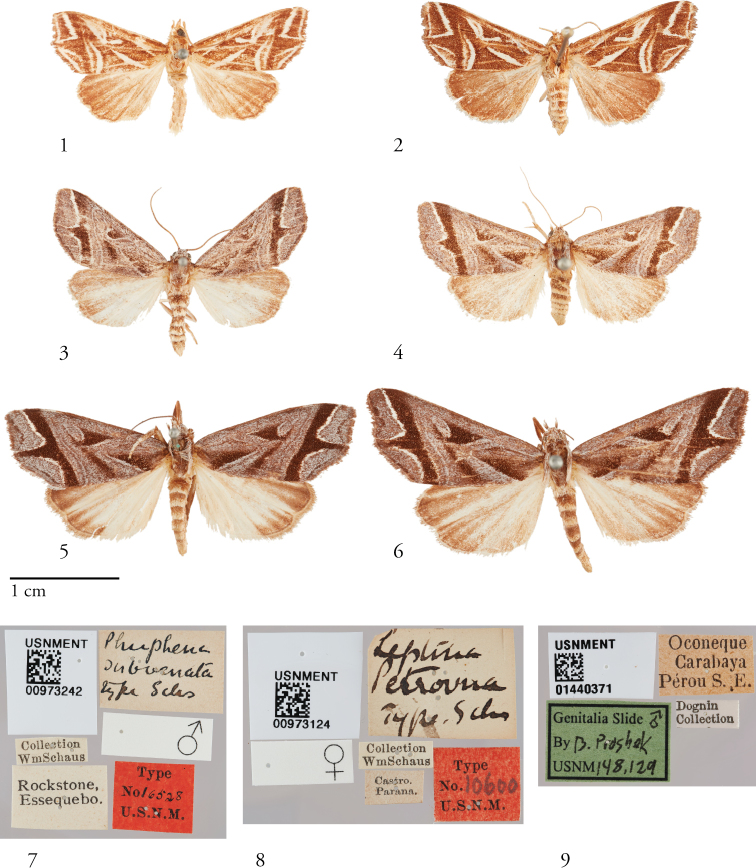
Dorsal habitus of *Thraumata* species. **1***T. subvenata* Holotype ♂, Guyana, USNMENT00973242 **2***T. subvenata*, USNMENT01440366♀, Suriname **3***T. petrovna* ♂ USNMENT01440358, Brazil **4***T. petrovna* Holotype ♀ USNMENT00973124, Costa Rica **5***T. peruviensia* Holotype ♂ USNMENT01440371, Peru **6***T. peruviensia* ♀ USNMENT01440374, Peru **7–9** Holotype specimen labels of *Thraumata* species **7***Phuphena subvenata* Schaus **8***Leptina petrovna* Schaus **9***Thraumata peruviensia* sp. nov.

#### Diagnosis.

Species of *Thraumata* are readily distinguished from *Phuphena* and *Callopistria* by the fragmented appearance of forewing pattern elements, the differential coloration of the hindwing upperside, paler towards the base with more darkly shaded margins, and the configuration of male and female genitalia, as follows: Valves without clasper complex (as in *Phuphena*, *Callopistria*, and *Phosphila*), but tapered and not clublike, unlike *Phuphena*, and with no differentiated cucullus or corona. Vinculum squared, less V-shaped than in *Phuphena*, extending below the valves for a distance less than the width at its broadest point. Vesica with multiple basal lobes and without cornuti. Ventrally recurved basal abdominal brushes present in *subvenata*; saccular coremata present and, except in *subvenata*, less developed than in *Callopistria*. The female genitalia are distinct, the corpus bursae without signa, elongate and bowed, not lemon-shaped as in *Phuphena*, the ductus bursae attaching postero-dorsally to the corpus, and the ductus seminalis arises from the posterior end of the corpus as in *Callopistria juventina*, not at the anterior apex as in all known *Phuphena* and several *Callopistria* species.

#### Description.

***Head***. Frons fully scaled; eye large, hairless; antenna filiform, shortly setose-ciliate in both sexes; labial palpus slightly upturned and densely scaled, third segment reduced compared to the first and second segments; proboscis well developed. ***Thorax***. Vestiture variously brown or grayish-brown scaling intermixed with white. ***Wings.*** Forewing elongate, not broadly rounded, the apex acute and the outer margin slightly angled at the middle; distal field crossed by conspicuous dark fascia outwardly produced at middle with triangular projection. Hindwing elongate too, slightly produced in correspondence to vein M3. Sexual dimorphism most obvious in hindwing, female with slightly darker shading towards wing base than male, but this character is neither discrete nor reliable. Underside of both wings brown or grayish-brown, the most prominent marking that of the toothed forewing submarginal line; Hindwing with 1–3 elongate pale streaks. A clearly expressed M2 visible on hindwing, arising from the discoidal cell closer to M3 than in *Callopistria*, possibly representing an autapomorphic condition for *Thraumata*. ***Legs.*** Outwardly pale, silvery white in *petrovna* and *peruviensia* sp. nov.; otherwise brown or with a mixture of brown and whitish scales. Foretibial epiphysis rugose; a single pair of striped mid-tibial spurs, two pair on hind-tibia; 3 rows of tibial spines on all legs. ***Abdomen***. Vestiture usually paler than on thorax and concolorous with hindwing surface; abdominal segments typically pale distally, more darkly banded at the anterior end of each segment, with darker scaling more uniformly diffuse below; typical trifine brush organs absent except in *T. subvenata* comb. nov., which bears a pair of tufts on male sternum A2, without pockets or levers; other species exhibit raised lateral flanges on sternum A2; posterior margin of 8^th^ sternite incised; rods extending from base of sternum A8 to pleurae. ***Male genitalia***. Tegumen raised at base of uncus; vinculum short relative to tegumen; paratergal sclerite visible but fused with tegumen. Valve tapered, not extending beyond tegumen, finely setose throughout and most heavily towards base. Uncus setose, swollen apically, terminating in a small point. Juxta tightly joined with valve and transtilla. Phallus asymmetrically sclerotized, narrow in its basal half; vesica with multiple bubble-like subbasal diverticula and a weakly to heavily sclerotized para-basal plate or lip from the distal end of the phallus; fremen of spermatophore with a well-developed nipple. ***Female genitalia***. Ductus bursae short, membranous, without colliculum; appendix bursae present at posterior end of corpus bursae, but not well differentiated; ductus seminalis arising from appendix bursae; corpus bursae oblong, without signa. Posterior apophyses rodlike, distally modified faintly, if at all. The bowed, ventrally facing configuration of the intersegmental membrane between A8 and A9 deformed so as to orient the corpus bursa at an angle with respect to the ventrally faceing papillae anales.

#### Immature stages.

Unknown.

#### Distribution.

Recorded from northern South America, southeastern Brazil, southern Peru, Bolivia, and Argentina along the eastern Andean slopes (Fig. 16).

### Key to species of *Thraumata* based on habitus

**Table:** 

1	Ground color of FW predominantly dark brown, all markings conspicuously highlighted in silvery white	*** T. subvenata ***
–	FW dominated by gray, with dark brown fascia in distal field bearing outwardly produced tooth, white lining confined to outer edge of this fascia (submarginal line)	**2**
2	FW length less than 13 mm (males) and 14 mm (females), toothed projection not reaching termen	*** T. petrovna ***
–	FW length greater than 14 mm (males) and 15 mm (females), toothed projection extended to termen	*** T. peruviensia ***

### Thraumata petrovna

Taxon classificationAnimaliaLepidopteraNoctuidae

(Schaus, 1904) comb. nov.

ad5320a5-e7d1-59bc-8db8-9c2019a394b0

Figs 3 , 4 , 12 , 13 (habitus); Fig. 17 (wing venation); Figs 18–20 (male genitalia); Fig. 25 (male abdomen); Fig. 29 (female genitalia)

Leptina? petrovna Schaus, 1904: 152. Type locality: Brazil [Rio de Janeiro], Petropolis. Figure: Hampson (1908: 597, pl. 121, fig. 23).

#### Material examined.

**Type material**. **BRAZIL**: **Holotype** ♀ (USNM). Collection Wm Schaus, Castro, Parana, Type No. 10600, *Leptina petrovna* Type. Schs, USNMENT00973124.

**Figures 10–15.**
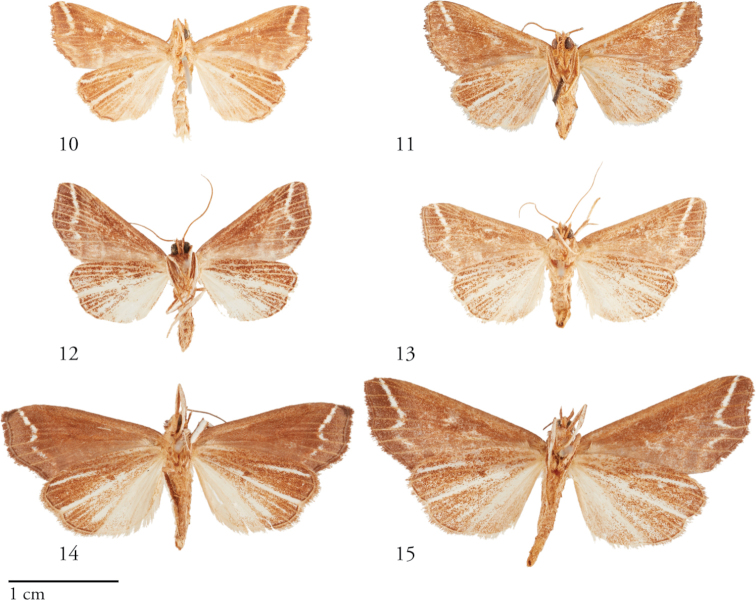
Ventral habitus of *Thraumata* species. **10***T. subvenata* Holotype ♂, Peru, NHMUK010606200 **11***T. subvenata*, USNMENT01440366 ♀, Suriname **12***T. petrovna* ♂ USNMENT01440358, Brazil **13***T. petrovna* Holotype ♀ USNMENT00973124, Costa Rica **14***T. peruviensia* Holotype ♂ USNMENT01440371, Peru **15***T. peruviensia* ♀ USNMENT01440374, Peru.

#### Other material examined.

**BRAZIL** (34♂, 39♀): **Paraná** (7♂, 7♀): *Males*: Banhado, Quatro Barras, Paraná, BRASIL – 800 m, 22.V.1971, Becker & Laroca, USNMENT01440489; Banhado, Quatro Barras, Paraná, BRASIL – 800 m 22.V.1971, Becker & Laroca; *Phuphena petrovna* (Schs, 1904), USNMENT01440447; Banhado, Quatro Barras, Paraná, BRASIL – 800 m, 27.II.1971, Becker & Laroca; *Phuphena petrovna* (Schs, 1904), USNMENT01440439; Castro. Parana.; *petrovna* Schs; Collection Wm Schaus, USNMENT01440466; Castro [ex coll. E.D. Jones] [NHMUK]; Castro, May 97 (E.D. Jones) [ex coll. Rothschild] [NHMUK]; Castro [ex coll. Joicey] [NHMUK]. *Females*: 2♀ Castro [ex coll. E.D. Jones] [NHMUK]; Castro, Febr. 97 (E.D. Jones) [ex coll. Rothschild] [NHMUK]; Castro, May 97 (E.D. Jones) [ex coll. Rothschild] [NHMUK]; Castro (E. D. Jones) [ex coll. Joicey] [NHMUK]; Castro. Parana.; *Leptina* [?] *petrovna* Schs Schs 99, USNMENT01440329; Becker Coll. No. 4573, Morumbi, Morrestes Paranà BRASIL 18.VII.1970 Becker & Laroca; *Phuphena petrovna* (Schs. 1904), USNMENT01440394. **Minas Gerais** (5♂, 3♀) *Males*: Campo bello Rio Zikan, USNM Dissection 148134, USNMENT01440385, BOLD Process ID LNAUW3136-17; Campo bello Rio Zikan; Noct 324 [illeg.] 15.4-31, USNM ♂ 41,204 SAB, USNMENT01440393; Campo bello Rio Zikan, Noct ♀ [sic] 324 [illeg.] 12.4-31, USNMENT01440370; [Ouro] Preto [ex coll. Joicey] [NHMUK]; Uberaba [ex coll. Le Moult] [NHMUK]. *Females*: BRASIL: MG 1400 m Serra do Cipõ 17–19.iv.1991, V.O. Becker coll., Becker Coll. No. 77884, USNMENT01440362; BRASIL: MG 1400 m Serra do Cipõ 17–19.iv.1991, V.O. Becker coll., Becker Coll. No. 77884, USNMENT01440368; BRASIL: MG 1400 m Serra do Cipõ 17–19.iv.1991, V.O. Becker coll., Becker Coll. No. 77884, USNMENT01440352. **Bahia** (1♀): BRASIL: BA Camacã, 400–700 m 21–30.ix.1991, V.O. Becker coll., Becker Coll. No. 83540, USNMENT01440364. **Santa Catarina** (7♂, 12♀): *Males*: Rio Vermelho, 830 m, June 1936 (A. Maller) [ex coll. Rothschild] [NHMUK]; Hansa Humboldt, 60 m, July 1936 (A. Maller) [ex coll. Rothschild] [NHMUK]; Brazil, Santa Catarina: Rio Vermelho VII-44 Anton Maller, USNMENT01463998 [AMNH]; Brazil, Sta. Catarina: Nova Teutonia, July 1–5 1951, Plauman, USNMENT01463709 [AMNH]; Brazil, Sta. Catarina: Nova Teutonia Plauman, USNMENT01463996 [AMNH]; The Sperry Collection, 23-7-48, Neuvo Teutonia, Brazil Plaumann coll., USNMENT01463920 [AMNH]; Rio Vermelho St. Cath., Brazil November 1947, dissection 148363, USNMENT01422625 [AMNH]. *Females*: Nova Bremen, 250 m, July 37 (F. Hoffmann) [ex coll. Rothschild] [NHMUK]; *idem*, Aug. 37, F. Hoffmann [ex coll. Rothschild] [NHMUK]; Neu Bremen, Rio Laeiss, Nov. 1935 (F.H. Hoffmann) [ex coll. Rothschild] [NHMUK]; *idem*, April 1936 (F.H. Hoffmann) [ex coll. Rothschild] [NHMUK]; Hansa Humboldt, 60 m, July 1936 (A. Maller) [ex coll. Rothschild] [NHMUK]; Becker Coll. No. 52409, BRASIL: SC, Scara, 300 m, 27.i.1983, V.O. Becker coll., USNM Dissection 148127, USNMENT01440363; Brasilien Nova Teutonia Fritz Plaumann, 27°80.0N Br. 52–53°Westl. L., 13, USNMENT01440353; Brazil, Sta. Catarina: Nova Teutonia July 12–17, 1948, F. Plaumann, USNMENT01463849 [AMNH]; The Sperry Collection, July 4, 1951, Neuvo Teutonia, Brazil Plaumann, coll., USNMENT01463774 [AMNH]; Collection of Grace H. and John L. Sperry Nova Teutonia Brazil, 24.7.48 Plaumann, USNMENT01463737 [AMNH]; Collection of Grace H. and John L. Sperry Brasilien Nova Teutonia, 27.7.1948, 87°11 [illeg.] 52°23, L. Fritz Plaumann, 300 [illeg.], 500 [illeg.], USNMENT01463784 [AMNH]; St Catherines Brazil, F. Johnson Collector, USNMENT01440404. **Espírito Santo** (1♀): USNMENT01440369 ♀. **Rio de Janeiro** (10♂, 7♀): *Males*: Rio (Derg.) [2♂ex coll. Rothschild; 1♂ ex coll. Joicey] [NHMUK]; 2♂ Rio Janeiro [ex coll. Walsingham] [NHMUK]; Rio de Janeiro, Organ Mts, near Tijuca / *Leptina petrovna* Schaus [ex coll. S. R. Wagner] [NHMUK]; 2♂ Petropolis [ex coll. Rothschild] [NHMUK]; Petropolis (M. Doer) [ex coll. Walsingham] [NHMUK]; Teresopolis, 13–22.iii.1957 [ex coll. Kettlewell] [NHMUK]. *Females*: Rio (Derg.) [1♀ ex coll. Rothschild; 1♀ ex coll. Joicey] [NHMUK]; Petropolis, 5-7-76 [ex coll. Joicey] [NHMUK]; Teresopolis, c. 2750 ft., 14–18.xii.57 (E. P. Wiltshire) [ex coll. Wiltshire] [NHMUK]; Pico d’Itatiaia, 28.iii–1.iv.1958 [ex coll. Kettlewell] [NHMUK]; Petropolis, Brazil.; *petrovna* Schs; Collection Wm Schaus, USNMENT01440482; Petropolis, Brazil.; *petrovna* Schs; Collection Wm Schaus; USNM Dissection 148128, USNMENT01440346. **Distrito Federal** (1♂): Becker Coll. No. 40221; Planaltina, DF. BRASIL-1000 m 15.V.1982, V.O. Becker coll. 15°35'S, 47°42'W; *Phuphena petrovna* (Schs, 1904), USNMENT01440358. **Goiás**: (1♀) Becker Coll. No. 20293; Formosa, Goiás BRASIL-800 m, 19.III.1977 V.O. Becker col., *Phuphena petrovna* (Schs. 1904), USNMENT01440386. **São Paulo** (2♂, 3♀): *Males*: São Paulo [ex coll. E.D. Jones] [NHMUK]; São Paulo [ex coll. Gerrard] [NHMUK]. *Females*: São Paulo (D. Jones); São Paulo [ex coll. Rothschild] [NHMUK]; São Paulo [ex coll. Gerrard] [NHMUK]. **ARGENTINA** (1♂, 3♀): *Male*: Argentina, Prov. Jujuy Dept. Ledesma 5–7.5 km W of Rt. 34 near entrance Parque Nacional Callilegua 1600 m, 14.II.1991; mesic forest along river, K. Johnson et al, USNMENT01463799 [AMNH]. *Females*: Argentina, Prov. Jujuy Dept. Capital; Baptist Mission[,] Camp at Rio Lozano, 1600 m, 28.II.1991; riparian woodland with steep forested slopes, K. Johnson et al., USNMENT01463997 [AMNH]; Argentina, Prov. Salta Dept. La Caldera; La Caldera–Jujuy Prov. border, Rt. 9, km 20, km post 1642: “La Cargadera”, 1450 m, 12.II.1991, mesic forest, K. Johnson et al., USNMENT01463797 [AMNH]; Argentina, Prov. Salta Dept. La Caldera; La Caldera–Jujuy Prov. border, Rt. 9, km 20, km post 1642: “La Cargadera”, 1450 m, 12.II.1991, mesic forest, K. Johnson et al., USNMENT01463840 [AMNH]. **BOLIVIA** (1♂, 1♀): Rio Songo, 750 m [via coll. Fassl ex coll. Oberthür] [NHMUK]; Rio Songo, 750 m [via coll. Fassl ex coll. Oberthür] [NHMUK]. **NO LOCALITY DATA** (1♂, 1♀): [no data] [ex coll. E.D. Jones] [NHMUK].

#### Diagnosis.

This and the following species are readily differentiated from *T. subvenata* by the gray-brown ground coloration and less prominent white markings throughout the forewing. *Thraumata petrovna* is, on average, the smallest of the three species, and although its wing pattern closely tracks that of the larger *peruviensia*, the postmedial tooth of the forewing is shorter, not reaching the wing margin; the vesica is distinctly more sclerotized, bearing a pronounced lateral band and a conspicuous, rugose plate.

#### Redescription.

***Head.*** Vertex and frontal scaling predominantly white but with a mixture of light gray-brown scales throughout the vertex, frons and palpi; frontal scales elongate. ***Thorax***. Vestiture a mix of white and grayish-brown scales. ***Wings.*** Forewing lengths: males 10.8–12.9 mm (*N* =13, *x*¯ = 12.2 mm, M = 12.5 mm); females (including holotype 12.1 mm) 11.0–13.7 mm (*N* =22, *x*¯ = 12.4 mm, M = 12.5 mm). Forewing ground color predominantly gray, with darker chocolate-brown buff in lower basal field, both below and between stigmata, and in toothed fascia of distal field; hindwing pale, brownish-gray towards margin; forewing and hindwing underside with diffuse chocolate-brown scaling, the shading on the hindwing concentrated between veins, fading towards hind margins on both forewing and hindwing; hindwing underside with a single conspicuous median pale streak. ***Legs.*** Outwardly pure white; otherwise light brown. ***Abdomen***. Abdominal segments with cream-colored scales distally, darker brown-banded at the anterior end of each segment; brown scaling more uniformly diffuse below. Abdominal brushes lacking. ***Male genitalia***. Tegumen rooflike, a distinct vertex and a pitch >45°. Sacculus bluntly rounded. Phallus heavily sclerotized, swollen and rugose along its outer edge; vesica with two rudimentary subbasal diverticula, and para-basal plate arising from distal end of the phallus. ***Female genitalia***. Posterior apophyses roughly 1.3× as long as anterior apophyses; papillae anales densely setose.

#### Immature stages.

Unknown.

#### Etymology.

Not explained, but evidently in reference to the type locality Petropolis (“City of Peter”).

#### Distribution.

Southeastern Brazil and along the eastern slopes of the Bolivian and Argentinian Andes.

#### Remarks.

A partial barcode sequence (658bp – 286n = 372bp) was generated from a Brazilian male specimen USNMENT01440385 corresponding to USNM dissection 148134 (Fig. 18), BOLD sample ID USNM_PG_H02. Schaus’ (1904) original placement of *petrovna* in what is now a synonym of the nolid genus *Baileya* may simply reflect the quadrifine appearance of its hindwing.

### Thraumata peruviensia sp. nov.

Taxon classificationAnimaliaLepidopteraNoctuidae

d3b78251-e701-5c3e-9bd0-9793b275d12c

http://zoobank.org/FBC0A9B5-7D7F-44F9-AFD9-5F3BC2B352F9

Figs 5 , 6 , 14 , 15 (habitus); Figs 21 , 22 (male genitalia); Fig. 26 (male abdomen); Fig. 28 (female genitalia)

#### Material examined.

**Type material**. **PERU** (11♂, 6♀): **Holotype** ♂ (USNM), Oconeque Carahaya Perou S. E.; Dognin Collection; USNMENT01440384. **Paratypes** (10♂, 6♀; USNM, NHMUK, AMNH): *Males*: same data as holotype, Dissection 148129, USNMENT01440371, BOLD Process ID LNAUW3137-17; same data as holotype, USNMENT01440341; Quinton, Carabaya, Peru, 5000 ft, i 1905. G. Ockenden [Joicey Bequest Brit. Mus. 1934-120] [NHMUK]; 2♂ Oconeque, Carabaya, Peru. 5000 ft, ii 1905, G. Ockenden; 1♂, Joicey Bequest Brit. Mus. 1934-120; 1♂, Rothschild Bequest B.M. 1939-1 [NHMUK]; 2♂ Oconeque, Carabaya, 7000 ft., dry s., July 1904 (G. Ockenden), Rothschild Bequest B.M. 1939-1 [NHMUK]; 3♂ Oconeque S. E. Peru, Feb’05 7000 ft, G. Ockenden [2♂, 67. 20. Ex Coll. Ed. Brabant 1920 / Rothschild Bequest B.M. 1939-1; 1♂ – 1909-89. [NHMUK]. *Females*: same data as holotype, Dissection 148130, USNMENT01440374; PERU: Cusco Machu Picchu 2885 m 5.II.1959 J.F.G. Clarke, USNMENT01440443; Macchu Pichu [sic] Peru II-11 1966 B. Heineman [AMNH]; Oconeque, Carabaya, Peru. 5000 ft, ii 1905, G. Ockenden; Joicey Bequest Brit. Mus. 1934-120 [NHMUK]; Oconeque, Carabaya, 7000 ft., dry s., July 1904 (G. Ockenden), Rothschild Bequest B.M. 1939-1 [NHMUK]; Oconeque S. E. Peru Feb’05, 7000 ft G. Ockenden, 67. 20. Ex Coll. Ed. Brabant 1920/Rothschild Bequest B.M. 1939-1 [NHMUK]

**Figure 16.**
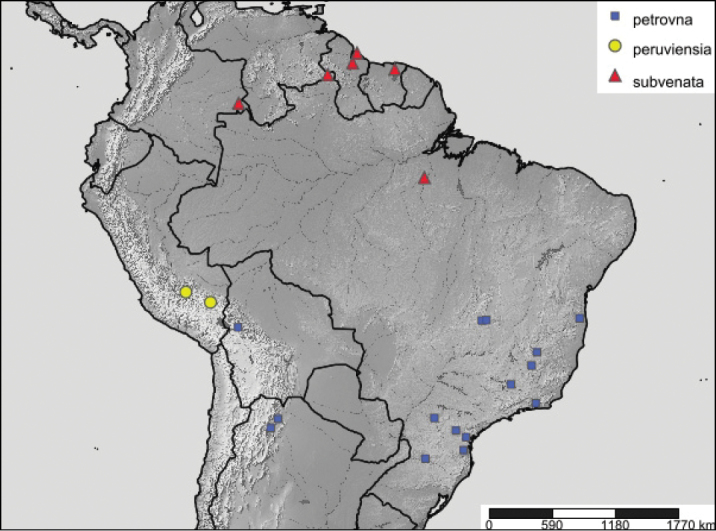
Known distribution of *Thraumata* species.

#### Diagnosis.

*Thraumata peruviensia* is most readily distinguished from the other two species by the combination of its larger size and distinctive vesica. In this and the previous species, the dominant ground color of forewing is gray, darker in the present species than in *T. petrovna*. This feature, the less pronounced white lining of pattern elements, particularly in basal and medial fields of forewing, and the comparatively reduced abdominal scent tufts in the male most readily distinguish this species pair from the more uniformly brown ground coloration of *T. subvenata*. In the new species the triangular tooth projecting from the dark brown distal fascia of the forewing is longer than in *T. petrovna*, and there are at least two distinct white streaks on the hindwing underside instead of one.

#### Description.

***Head.*** Vestiture as in *T. petrovna*. ***Thorax***. Vestiture a mix of grayish-brown scales intermixed with white, especially within tegulae. ***Wings.*** Forewing lengths: males (including holotype 15.3 mm) 14.2–15.3 mm (*N* = 3, *x*¯ = 15.0 mm, M = 12.5 mm); females, 15.5–16.8 mm (*N* = 3, *x*¯ = 16.1 mm, M = 16.1 mm). Forewing coloration dominated by gray scaling suffused with white, lines for the most part obscured, although in some individuals antemedial and postmedial lines still discernible as faint, thin pale gray lines. Four areas of more uniform, chocolate-brown scaling: an elongate, obtuse subtriangular smudge in lower basal field from wing base to antemedial line; an oblique band in place of median shading between from reniform stigma and inner margin, dark filling between orbicular and reniform stigmata, and in fascia bearing outwardly projected median tooth of distal field, this fascia lined externally with silvery white. Forewing underside nearly uniform chocolate brown excepting paler white inner marginal area; the shading on the hindwing underside interrupted by two-three conspicuous pale streaks, fading towards wing base. ***Legs.*** White outward, otherwise as brown as in previous species. ***Abdomen***. Abdominal segments predominantly gray, ringed with pale cream-colored scales distally; abdominal brushes lacking. ***Male genitalia***. Sacculus squared. Sclerotized lateral band on phallus less pronounced than in *petrovna*, vesica with three rudimentary subbasal diverticula and reduced para-basal plate from distal end of the phallus. ***Female genitalia***. Ductus bursae quite short relative to distended corpus bursae. Anterior and posterior apophyses roughly co-equal. Papillae anales densely setose. As in other *Thraumata*, the ductus seminalis arises from a bulge at the posterior end of the corpus bursae.

**Figure 17.**
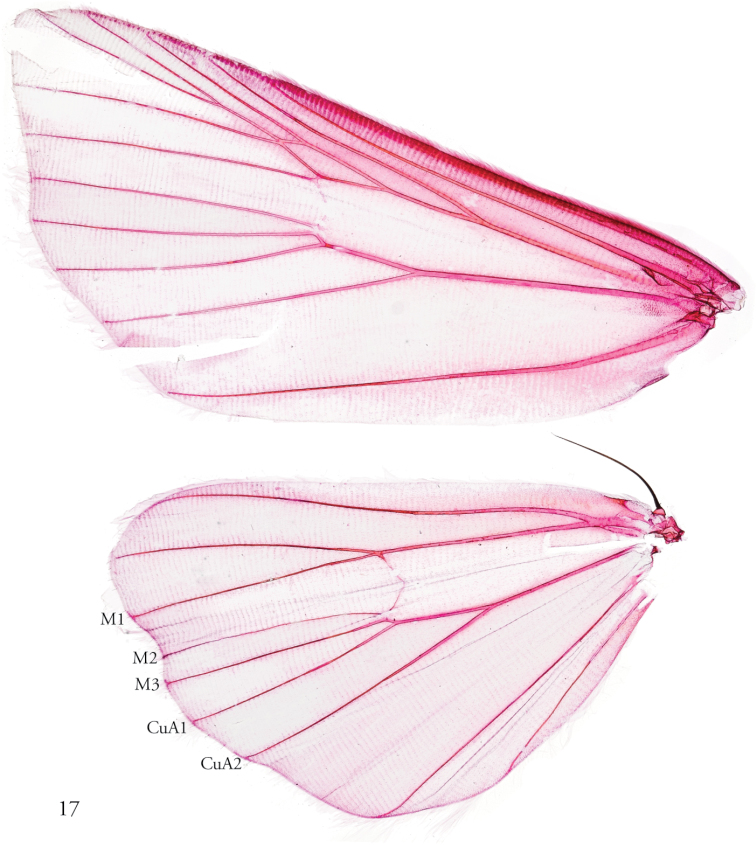
Wing venation of *T. petrovna*, male, USNMENT01440439, wing preparation 148365.

#### Immature stages.

Unknown.

#### Etymology.

The name *peruviensia* is a neuter pleural adjective, in reference to the country of origin of the new species, in coordination with the generic name *Thraumata*.

#### Distribution.

Southeastern Peru.

#### Remarks.

This species is very similar in appearance to *T. petrovna* but consistently larger and occurring at higher altitudes. An incomplete barcode sequence (658bp – 88n = 570bp) was generated from a 1905 male specimen, USNMENT01440371 corresponding to USNM dissection 148129 (Figs 21, 22), BOLD sample ID USNM_PG_H03.

**Figures 18–22.**
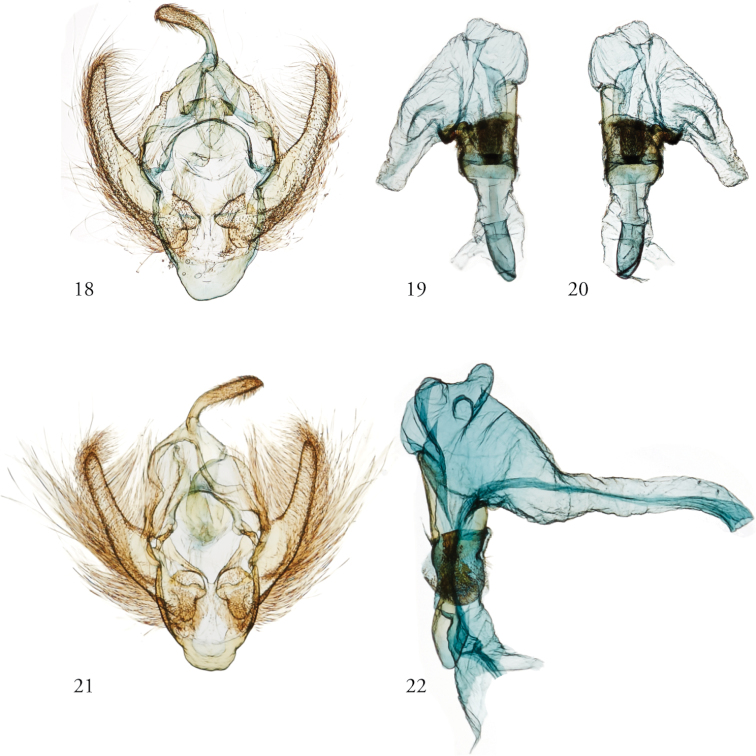
*Thraumata* male genitalia. **18–20***T. petrovna* dissection 148363, USNMENT01422625, Brazil **18** Apparatus **19, 20** Phallus **21, 22***T. peruviensia*, dissection 148129, USNMENT01440371 **21** Apparatus **22** Phallus.

### Thraumata subvenata

Taxon classificationAnimaliaLepidopteraNoctuidae

(Schaus, 1914), comb. nov. (provisional position)

78b797e2-064f-56ce-9809-e04dbcd93023

Figs 1 , 2 , 10 , 11 (habitus); Figs 23 , 24 (male genitalia); 27 (male abdomen); Fig. 30 (female genitalia); Figs 31–34 (morphological details).

Phuphena subvenata Schaus, 1914: 486. Type locality: [Guyana] British Guiana: Essequebo River, Rockstone.

#### Material examined.

**Type material**. **GUYANA**: **Holotype** ♂ (USNM). Collection Wm Schaus, Rockstone, Essequebo, Type 16528 USNM, *Phuphena subvenata* Type Schs, USNMENT00973242. **Paratype. GUYANA**: 1♂ Rockstone, Essequebo; *Phuphena subvenata* Schs, W. Schaus, 1912-38. [NHMUK];

#### Other material examined

(3♂, 7♀). **GUYANA**: 1♀ Demerara; Rothschild Bequest, B.M. 1939-1 [NHMUK]; 1♀ Demerara; 75; Adams Bequest. BM 1912-399., NHMUK010914717, slide NHMUK010314585 [NHMUK]; 1♂ Brit. Guiana, Kaialam [illeg.], 26.V.1929, [illeg.] T.D.A. Cockerell, Pres. by Imp. Bur. Ent. Brit. Mus 1930-188., NHMUK010914410, slide NHMUK010314636 [NHMUK]; 1♂ Georgetown Br. Guiana; July; A Busck coll, Not in BM 1925, USNM Dissection 148139, USNMENT01440389, BOLD Process ID LNAUW3138-17. **SURINAME** (1♀): Geldersland [sic], Surinam River.; Collection Wm Schaus, USNMENT01440366. **VENEZUELA** (1♀): Venezuela, Bolivar: Guiaquinima tepui, camp 1, 5°55'N, 63°30'W, 1150 m, Feb. 24–28, 1990, D. Grimaldi [AMNH]. **COLOMBIA** (1♂, 1♀): Ober Rio Negro, Ost. Colomb., 800 m., Coll. Fassl; Ex. Oberthür Coll. Brit. Mus. 1927-3.; (♂) NHMUK010201223; slide NHMUK010314637 [NHMUK]. **BRAZIL** (2♀): Pará (A. M. Moss); Rothschild Bequest, BM, 1939-1, NMHUK010916076 [NHMUK].

#### Diagnosis.

The predominantly golden-brown coloration of the forewing upperside and the prominent white edging of its markings readily distinguished this species from *T. petrovna* and *T. peruviensia*. Valvae are more broadly articulated with the vinculum than in the other species, the costal edge <1/2 the length of the outer edge. Unlike the two predominantly gray-brown species, *T. subvenata* also bears basal abdominal brushes and what appear to be well-developed eversible coremata on the sacculus.

**Figures 23, 24.**
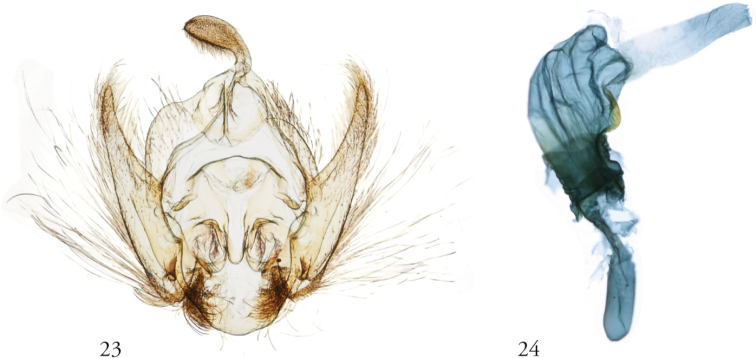
*Thraumata subvenata***23** Apparatus, dissection 148139, USNMENT01440389 **24** Phallus, NHMUK010201223, slide NHMUK010314637, Colombia.

#### Redescription.

***Head***. Proboscis edged with papillae and a micro-serrated ridge. Antennae ciliate; eyes hairless; palpi extending above eyes. Frons, vertex and palpi with a mix of brown and white scales; terminal segment of the palpus whitish. ***Thorax***. Vestiture predominantly light brown, a whitish V formed by two lines of whitish scales within the tegulae. ***Wings.*** Forewing length, males (*N* = 2), 11.5 (holotype)–12.0 mm; females (*N* = 3), 12.1 mm, 12.4 mm, 13.0 mm. Forewing coloration dominated by golden-brown scaling; the differential white shadowing, of various pattern elements, particularly the postmedial and subterminal lines, renders a fractured appearance consisting of streaks and sickle-shaped forms: two basal white streaks, 2 crescent-shaped medial markings bisected by brown inner line and two, more expansive, subterminal forms, each enclosing a brown center. These latter four simply bracket the broad, dentate subterminal band homologous to that in the previous two species. ***Legs.*** Predominantly brown, paler outwardly but not silvery white. Male fore-femur with a thick, cream-colored pencil of long scales (Figs 31, 32), absent in females and therefore likely a scent tuft. ***Abdomen***. Posterior edges of abdominal segments ringed with cream-colored scales. Males with several secondary sexual structures, including distal scale tufts borne by the lateral rods of 8^th^ sternite and a pair of mixed gray and brown abdominal brushes on A2 sternite, visible in situ on the holotype as arching inward and overlapping above the midline (Fig. 33). The holotype was clearly damaged and repaired poorly, with abdomen re-attached upside down, such that ventral brushes appear dorsal. Tufts of elongate hairs present on 8^th^ sternite (Fig. 34). ***Male genitalia***. Relative to the rest of the moth, valvae the largest of the three species; costal and ventral edges swollen to form an inner crest; a bundle of stiff hairs inserted on the infero-lateral corners of valvae; valvae fused to vinculum along more than half their length. Vesica with three rudimentary subbasal diverticula, para-basal plate at the distal end of the phallus weakly sclerotized, appearing faintly crenulate. ***Female genitalia***. Anterior and posterior apophyses comparable in length. Antrum well developed, cuplike. Relative to its congeners, *T. subvenata* has a longer ductus bursae, with the ductus seminalis arising from a bulge along the ductus bursae, posterior to the corpus.

#### Immature stages.

Unknown.

#### Distribution.

Known only from northern South America, specifically Colombia, Venezuela, Guyana, Suriname and Brazil (Pará).

#### Remarks.

Both the valvae and the legs of *subvenata* males are equipped with more conspicuous scent tufts than either of the two sister species. An incomplete barcode sequence (658bp – 88n = 570bp) was generated from a 1925 male specimen from Guyana USNMENT01440389 corresponding to USNM dissection 148139 (Figs 21, 22), BOLD sample ID USNM_PG_H04.

**Figures 25, 26.**
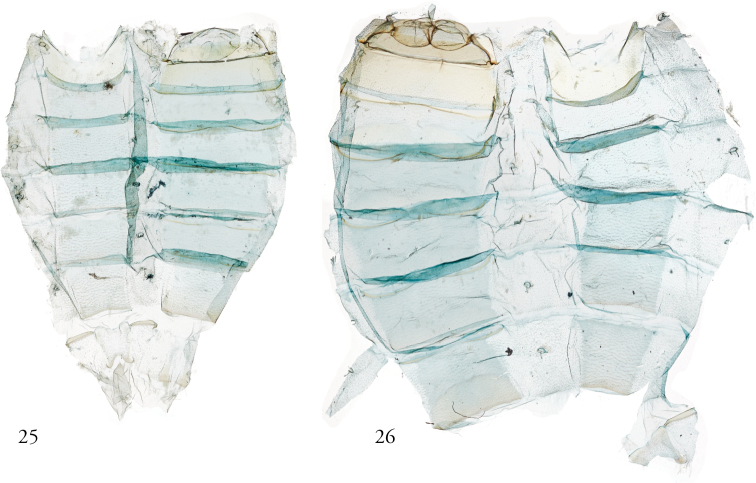
*Thraumata* male abdomens. **25***T. peruviensia* dissection 148134, USNMENT01440385 **26***T. peruviensia* dissection 148129, USNMENT01440371, Brazil.

**Figure 27.**
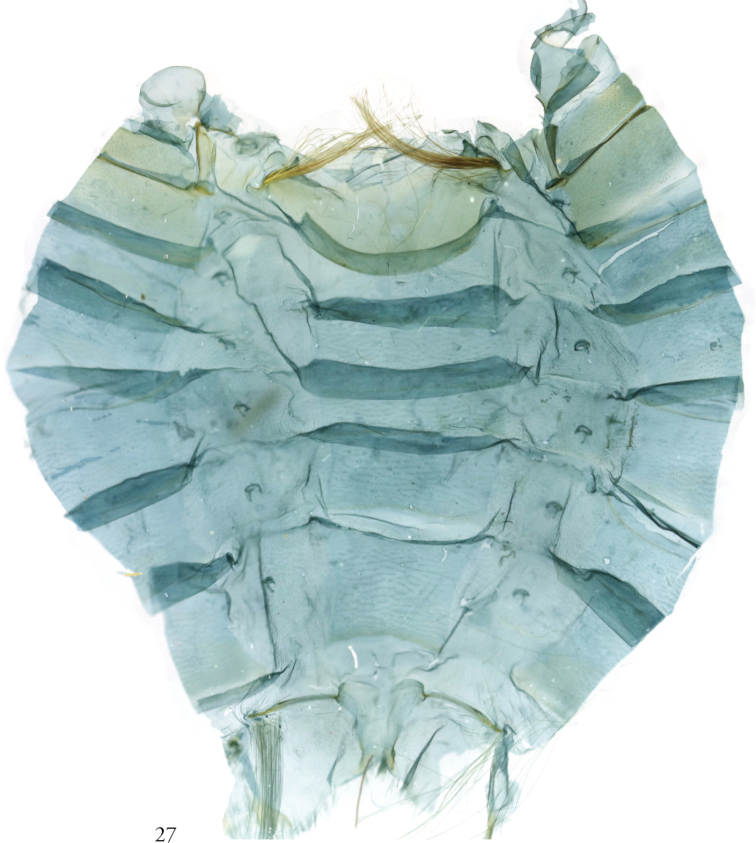
*Thraumata subvenata* NHMUK010201223, slide NHMUK010314637, Colombia.

## Discussion

The boundaries of the Eriopinae have yet to be established as several of the characters used to circumscribe the subfamily are either widespread or have been characterized vaguely, and are likely to be revisited during the course of ongoing studies. For present purposes, we suggest the possibility that placement of *Thraumata* within the Eriopinae on the basis of characters shared by *Callopistria* (= *Eriopus* Treitschke, 1825). These include the tapered, ear-shaped valves without a linear corona, the presence in *T. subvenata* of small saccular coremata on the inner face of the valves, and narrow sclerotization along the basal half of the phallus, which dissipates in the distal third (cf. Holloway 1989; Yen and Wu 2009). The “ballooning” of the tegumen noted in several *Callopistria* can also be seen, particularly in *T. subvenata* (Fig. 23), which is the only *Thraumata* with basal abdominal hair pencils (Fig. 27) known in most *Callopistria* species but absent from Phosphilini. The configuration of the male genital capsule of *Thraumata* differs sharply from that of *Phuphena*, in which a uniformly slender stalk-like valve devoid of basal coremata ends in a clubbed cucullus with a diffuse corona of stout setae. *Thraumata* and *Phuphena* share with *Callopistria* conspicuous tufting of the dorsal side of valves, but differ in the position of the ductus seminalis with respect to the bursa copulatrix (posterior in *Thraumata* and anterior on the fundus bursae in *Phuphena*; this character varies in *Callopistria* as noted above), and in the oblique orientation of the orbicular and reniform stigmata towards one another in *Thraumata*. *Thraumata* may also be differentiated from *Callopistria* by the short incrassate uncus and, at least in the case of the species pair *petrovna* and *peruviensia*, by elongate wings and the origin of hindwing M2 in close proximity to M3. In addition to the developed saccular and abdominal coremata, several features of *T. subvenata* are sufficiently reminiscent of *Callopistria* to consider either its placement in *Thraumata* tentative, or the possibility that the whole of *Thraumata* are phylogenetically embedded subgenerically within *Callopistria*. These include the configuration of the juxta, with its deep mesial invagination and broad lateral plates; less elongate wings than in *T. petrovna* or *T. peruviensia*; and the position of M2 further from M3 than in the other two species of *Thraumata*. The position of the ductus seminalis of *T. subvenata* on the posterior side of the corpus bursae is similar to that of other *Thraumata*, but differs in that it arises from a bulge emerging from the ductus bursae itself, not from the corpus. Both these configurations differ from that of *Phuphena*, in which the ductus seminalis arises from the distal end of the corpus. In *Callopistria juventina*, the ductus seminalis is described as arising from sclerotized wrinkles on the posterior end of the bursa (Fibiger and Hacker 2007: 51), but in our observations the area in the immediate vicinity of the base of the ductus seminalis is membranous, and variants of all these character states may be observed elsewhere in the genus (e.g., *C. phaeogona* Hampson, 1908; cf. Yen and Wu 2009:28, Fig. 14B).

**Figures 28, 29.**
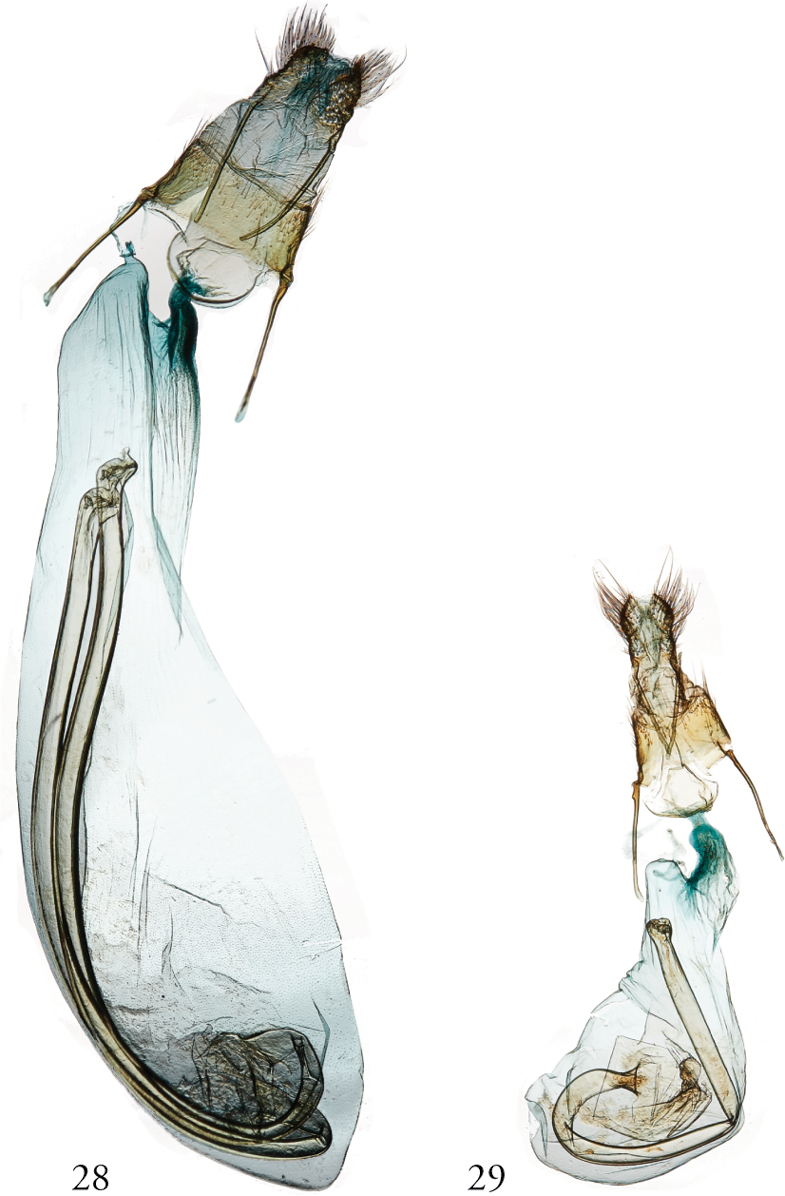
*Thraumata* female genitalia. **28***T. peruviensia* dissection 148130, USNMENT01440374 USNM, Brazil **29***T. petrovna* dissection 148127, USNMENT01440363, Peru.

**Figure 30.**
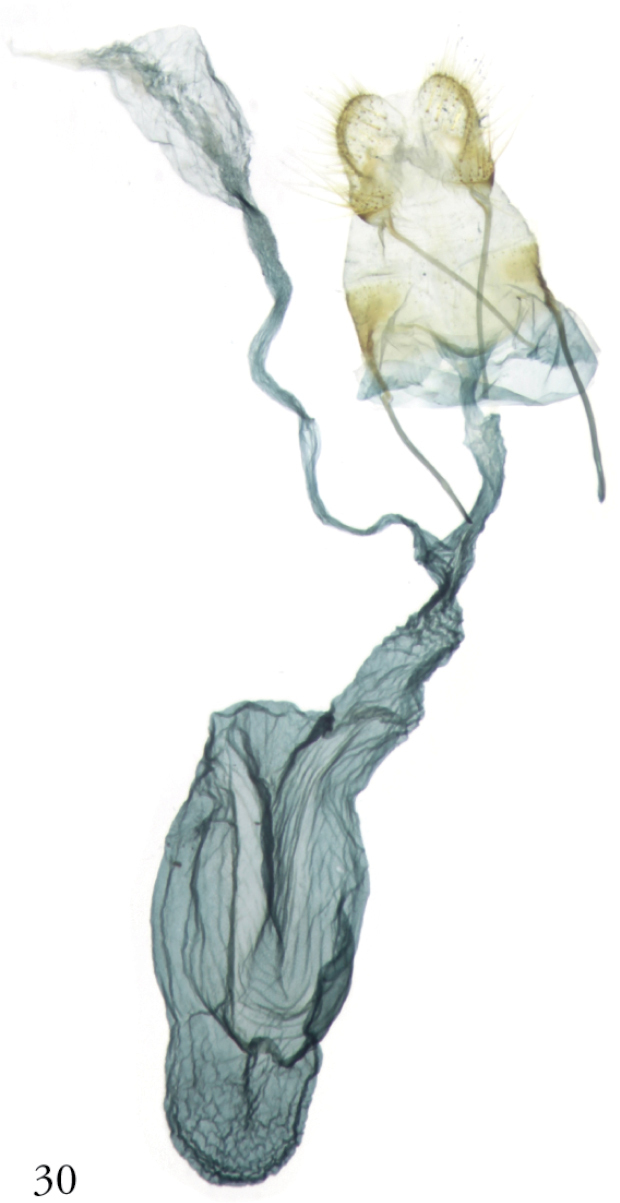
*Thraumata subvenata*, female genitalia. NHMUK010914717, Guyana.

The pleural tufts on A8 of *T. subvenata* (Fig. 27) are absent from its congeners, although in these the rods remain expressed. Such variation among close relatives is not unusual or inconsistent with the lability of secondary sexual characters elsewhere in the Noctuidae (e.g., Apameini, Phlogophorini) or elsewhere in the Lepidoptera (e.g., Pyraloidea, cf. Goldstein et al. 2013). The hair pencils on A2 of *T. subvenata* (Figs 27, 33) are consistent with Fibiger and Lafontaine’s (2005: 41) statement to the effect that such brushes in Eriopinae are “thought not to be homologous with [those] of other noctuids,” since they lack typical pockets and apodemes. They are not, however, divided into sub-pencils or otherwise consistent with Poole’s (1995: 18) description of eriopine abdominal brushes. The more diffuse tufts at the base of the valves on *T. subvenata* (Fig. 23) are comparable to the eversible saccular coremata mentioned by Fibiger and Lafontaine as well as Holloway (1989) as features of Eriopinae. Other such features adduced by Yen and Wu (2009) and visible in *Thraumata* include the “ladder-shaped” (or H-shaped) antero-medially incised male 8^th^ abdominal sternite; scale tufts associated with the paired pleural rods at the base of sternum A8 (only in *T. subvenata*, Fig. 27); the absence of the clasper complex from the valve (Figs 18, 21, 23); short vinculum (especially in *T. subvenata*, Fig. 23); narrow, asymmetrically sclerotized phallus (Figs 19, 20); and, in the female, a membranous, oblong corpus bursae (Figs 28–30). None of these can be considered uniquely derived within Noctuidae, however.

Fibiger and Lafontaine (2005: 41) placed *Phuphena* in Phosphilini based on the simple valve and spiny vesica, but this assignment may be problematic. Although the vesica of *Phosphila turbulenta* Hübner, 1818 (the type species of *Phosphila*) is similar in shape to those of *Phuphena*, it is spineless. Where vesical spines do occur *Phosphila* (e.g., in *P. dogmatica* Dyar, 1916), they are fine and enclose the basal portion of the vesica. Spines on the vesicae of *Phuphena*, on the other hand, are confined to a ventral band of scale-like cornuti. The valves of typical *Phuphena* are stalk-like, swollen or slightly swollen apically, whereas those of *Phosphila* are more expansive and gradually tapered. *Phuphena* species do share a number of taxonomically widespread features (or losses thereof) with Phosphilini, including naked, lashless eyes, and elongate, membranous corpus bursae without signa. Similarities in the male genitalia include the absence of a free pleural sclerite, the valves simplified in such a way that they are without clasping architecture, and the corona (if so termed) comprises an apical, unarranged concentration of setae (cf. “hairlike setae”; Poole 1995: 21). The dimensions of *Thraumata* valvae are more similar to those of *Callopistria* than *Phosphila*, i.e. tapered and not narrowed throughout or clublike. The phalli of *Thraumata* share the pattern of sclerotization seen in both *Phuphena* and *Callopistria*. Although we hasten to add that both *Phuphena* and *Thraumata* are significantly smaller-bodied than most Phosphilini, they do not bear any immediately obvious similarities with *Acherdoa ferraria*, which possesses highly reduced male genitalic features. As Poole (1995: 21) described, adult Phosphilini also lack basal abdominal hair pencils of any kind, and although this character is notoriously homoplastic throughout the Noctuidae, and appears polymorphically in both *Thraumata* and *Callopistria*, its absence is consistent throughout Phosphilini, and its presence consistent among all known *Phuphena*.

DNA barcode data, including partial sequences of the 658bp COI barcode region obtained for all three species of *Thraumata*, provisionally support the monophyly of both *Phuphena* and *Thraumata* and unite them with the eriopine genera *Callopistria* and *Argyrosticta*. But these results must be viewed skeptically, given their incompleteness (570bp for both *T. peruviensia* and *T. subvenata*, and 372bp for *T. petrovna*; see Material examined above for BOLD process ID codes of available sequences). Although the larvae of *Phuphena* have thus far been associated exclusively with ferns and differ in many other respects from the gaudy, semi-gregarious larvae of Phosphilini, which have been recorded only from Smilacaceae, we have as yet only been able to examine larvae indirectly, through images. Without specimens, none of the larval characters discussed by Crumb (1956), Beck (1960, 1999–2000), Poole (1995), Fibiger and Lafontaine (2005), or Yen and Wu (2009) that might support or refute their placement in Eriopinae can be examined critically, and to our knowledge no reared larvae of *Thraumata* have been described or imaged. These circumstances reinforce the need for a thorough revision of the current concept of the Eriopinae as the analyses of groups long placed in the Amphipyrinae proceed (Keegan et al. 2019). While we find it likely that the taxonomic diversity of New World Eriopinae is broader than previously supposed, a more precise circumscription of the group will ultimately depend on a combination of additional larval information and molecular phylogenetic data.

**Figures 31–34.**
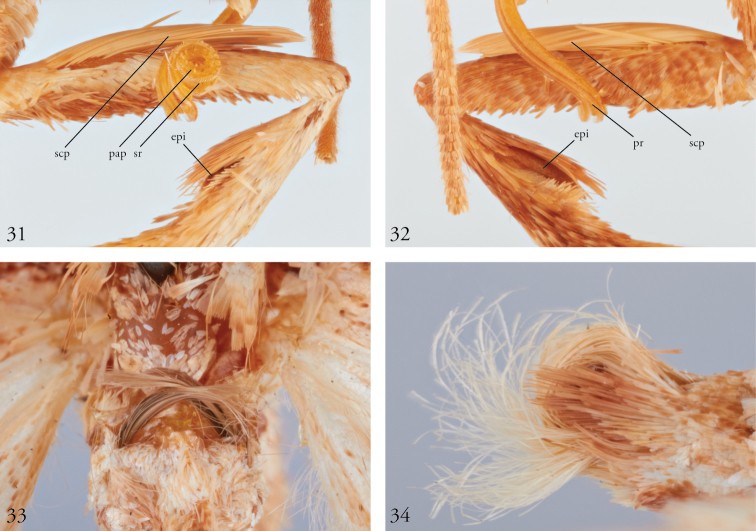
*Thraumata subvenata*, holotype (male), foreleg, proboscis, and abdominal structures. **31** Right foreleg, outside lateral view, indicating femoral scale pencil (sp), epiphysis (ep), and proboscis (pr) with papillae (pap) and serrated lateral ridge (sr) **32** Right foreleg, inside lateral view, indicating femoral scale pencil (sp) and epiphysis (ep) **33** Ventral brushes on A2 **34** Abdominal terminus, showing distal scale tufts.

## Supplementary Material

XML Treatment for Phuphena

XML Treatment for Thraumata

XML Treatment for Thraumata petrovna

XML Treatment for Thraumata peruviensia

XML Treatment for Thraumata subvenata
